# An Optimal Schedule for Urban Road Network Repair Based on the Greedy Algorithm

**DOI:** 10.1371/journal.pone.0164780

**Published:** 2016-10-21

**Authors:** Guangquan Lu, Ying Xiong, Chuan Ding, Yunpeng Wang

**Affiliations:** 1 School of Transportation Science and Engineering, Beijing Key Laboratory for Cooperative Vehicle Infrastructure System and Safety Control, Beihang University, Beijing, 100191, China; 2 Jiangsu Province Collaborative Innovation Center of Modern Urban Traffic Technologies, Si Pai Lou #2, Nanjing, 210096, China; Tianjin University, CHINA

## Abstract

The schedule of urban road network recovery caused by rainstorms, snow, and other bad weather conditions, traffic incidents, and other daily events is essential. However, limited studies have been conducted to investigate this problem. We fill this research gap by proposing an optimal schedule for urban road network repair with limited repair resources based on the greedy algorithm. Critical links will be given priority in repair according to the basic concept of the greedy algorithm. In this study, the link whose restoration produces the ratio of the system-wide travel time of the current network to the worst network is the minimum. We define such a link as the critical link for the current network. We will re-evaluate the importance of damaged links after each repair process is completed. That is, the critical link ranking will be changed along with the repair process because of the interaction among links. We repair the most critical link for the specific network state based on the greedy algorithm to obtain the optimal schedule. The algorithm can still quickly obtain an optimal schedule even if the scale of the road network is large because the greedy algorithm can reduce computational complexity. We prove that the problem can obtain the optimal solution using the greedy algorithm in theory. The algorithm is also demonstrated in the Sioux Falls network. The problem discussed in this paper is highly significant in dealing with urban road network restoration.

## Introduction

Research related to the road network reconstruction plan for earthquakes, floods, and other catastrophic events has noticeably increased over the past decade. Although these events are undeniably important, small daily life events should not be ignored. A wide variety of traffic accidents, car break down, road maintenance, storm-water ponding, road deterioration, and bad weather will cause partial or total reduction in capacity on a given link of urban road network. Traffic congestion, increase in road network travel cost, and even a gridlock can happen if these links are not repaired and their capacity are not restored in time. For example, during a heavy rain in Beijing in July 21, 2012, the capacity of the 95 road sections of the urban network became zero, and the storm caused a traffic gridlock. This event still has profound effects on the urban road network despite it being relatively “minor” compared with catastrophes. On-time road network repair is urgent. However, the critical link should be identified when repair resources are limited. Accordingly, identifying which link or links will be given priority during repair becomes particularly important. An appropriate schedule should be prepared based on this information.

Critical links have varying definitions for different researchers or objectives. Corley and Sha [1] proposed that the most vital links in a weighted network would be those whose removal from the network would result in the greatest increase in the shortest distance between two specified nodes. Nardelli et al. [2] studied the difference between the length of the detour path after any link interrupted in the shortest path and that of the original shortest path to measure link importance. Scott et al. [3] defined the network robustness index (NRI) to identify critical links. The NRI is substantially equal to the change in the network-wide travel time when a given link is removed from the network. Oliveira et al. [4] pointed out that using congestion and vulnerability to acquire the importance ranking of road network links was appropriate. Rupi et al. [5] ranked network links according to their importance in maintaining proper connectivity among all origin–destination pairs. Hou and Jiang [6] proposed an indirect method to evaluate the relative importance of a link by using link reliability importance. Sohn [7] suggested that the accessibility index could be used to evaluate the significance of highway network links under flood damage. Current studies have identified the critical link mostly by considering the destruction or removal of a link. The link, which is vital for road network robustness, is not necessary for road network restoration. Therefore, we define critical link from the perspective of road network restoration. Our research focuses on how much the restoration of a link can contribute to road network performance in evaluating the critical link. Meanwhile, we also do not ignore the fact that a road network is dynamic. That is, evaluating the critical link is dynamic.

In recent years, complex networks have been studied widely related to the properties and application of complex networks [8–10]. It will work well based on a good robustness for the network [11]. As to the complex road network, the studies on the network robustness mostly focus on dealing with disasters so far. Studies on dealing with disasters can be divided into two categories as follows: 1) enhancement of vital facilities to increase network robustness before a disaster happens and 2) quick response after a disaster. With regard to enhancing network robustness, the main research objective is to allocate limited resources to enhance vital facilities and reduce loss during a disaster. Protection and planning for recovering vital network segments are an efficient proactive approach to reduce the worst-case risk of service disruption because of budgetary limitations [12]. On the basis of such consideration, exploring the vulnerability of network nodes or arcs to disruption [13] and establishing the bi-level program model to protect the critical network segment to respond to attacks are the main research objectives [14, 15]. Most of the research background for network reconstruction and emergency rescue is disaster. The core of these studies is the effectiveness of limited resource allocation. Giving priority to the important edges which connected nodes with the largest populations is an effective repair strategy [16]. In addition, there are various measure indicators to help allocate resources. The effectiveness of limited resource allocation can be measured by minimizing system cost and maximizing system flow [17]; maximizing network accessibility [18]; minimizing user travel costs [19]; minimizing the rescue costs of primary and secondary disasters [20]; maximizing cumulative network accessibility and minimizing make span [21]; optimizing accessibility [22]; minimizing the travel time of travelers, total working time, and idle time between work troops [23]; minimizing combinatorial indicators [24]; maximizing the performance of emergency rehabilitation; minimizing the risk of rescuers and maximizing the saving of lives [25]; and minimizing unsatisfied demands for resources, time to delivery, and transportation costs [26] among others.

Protecting the critical network segment is vital before random or deliberate attacks. However, maintaining normal service is insufficient most of the time, which means that we should also quickly respond after network incidents occur. Moreover, we must recover its service on time. Most research objectives focus on disasters. Accordingly, the vehicle routing model is the core of these studies, and considerable constraints that should be solved optimally are involved in the model. In this study, we focus more attention on repairing damaged road networks resulting from minor events. We aim to minimize the cumulative whole network travel cost when we only have one repair crew (repair crew can be expanded). We propose the road network repair schedule-based greedy algorithm, which significantly improves computational efficiency, based on critical link identification. We can quickly obtain the optimal urban road network schedule even if the road network is extremely large. We prove that the greedy algorithm can obtain an optimal solution for our problem in theory. The test results show that an optimal schedule can be efficiently derived by our greedy algorithm.

The rest of this paper is organized as follows. Section 2 introduces the definition of the critical link and the optimal schedule for urban road network repair based on the greedy algorithm. Proof is also provided in this section. Section 3 tests the developed road network repair crew scheduling in the Sioux Falls network and presents the analysis results. Section 4 concludes the study.

## Methodology

This study focuses on an optimal urban road network repair crew scheduling. The repair crew can only repair links when the capacity of some urban road network links is destroyed because of various reasons, and we only have one repair crew. Our research aims to minimize the cumulative whole road network travel cost along with damaged link restoration. A different repair order certainly results in a different effect in urban road network performance. The exhaustive search method requires a large calculation workload. Moreover, link restoration may worsen road network situations because of the Braess’ paradox. A greedy algorithm is an algorithm that applies the problem-solving heuristic of making a locally optimal choice at each stage with the aim of finding a global optimum. This algorithm performs efficiently for certain scheduling problems [27, 28]. We propose the optimal schedule for an urban road network repair based on the greedy algorithm because of its advantages. This algorithm aims to quickly obtain an optimal schedule, thereby ensuring that the effort of the repair crew will result in efficacious network improvement during the repair process. We also prove that the greedy algorithm is applicable to our problem in theory. Although our study is more theoretical rather than practical, it retains the basic characteristics of traffic. The result can still guide the repair of urban road networks in real life.

In early studies, scholars used to represent link damage with a 100% capacity reduction on the link. The most obvious problem resulting from such approach is the creation of isolated sub-networks. Moreover, a complete link from the network is not associated with reality. Several scholars have considered that using a high percentage-based link capacity reduction instead of 100% can be better. Sullivan et al. [29] extensively investigated this problem. The result showed that the most stable capacity disruption range for the ranking of critical link varied with network connectivity level. Consistent with the literature, the damaged links in our research indicate a high percentage-based link capacity reduction. The capacity reduction will be determined using road network connectivity.

### Parameters

The critical link will be initially repaired in our greedy algorithm. Therefore, this part will introduce the definition of the critical link. In this study, we focus on the ratio of the travel cost in different network states, rather than on the specific travel cost, to facilitate comparison. The link whose restoration produces the ratio of the system-wide travel time cost of the current network to the worst network is at minimum. We define such a link as the critical link for the current network. The notations listed in Table 1 have been adopted to facilitate description.

**Table 1:** Notation description.

Notation	Definition
*E*	Set of all links in the road network
*E*_*normal*_	Set of normal links in the road network, abbrev *E*_*n*_
*E*_*repair*_	Set of abnormal links in the road network, abbrev *E*_*r*_
*E*_*ni*_	Set of normal links before repair the *i*-th link in the road network
*E*_*ri*_	Set of abnormal links before repairing the *i*-th link in the road network
*C*_0_	System-wide travel cost in the initial state
$c_{e}^{i}$	System-wide travel cost after repairing *i* links and link e is repaired in the last
$I_{e}^{i}$	Ratio of $c_{e}^{i}$ to $c_{e}^{i}$, it represents the importance of a given link *e*
*i*	*i* = 1,2,3,…,*m*; m is equal to the number of links belonging to *E*_*r*_

First, we calculate *c*_0_ and *c*_0_ as the base of all calculations. *c*_0_ can be calculated as follows:

$$c_{0}=\sum_{j\in \left\{E_{n1},E_{r1}\right\}}t_{j}x_{j},$$ (1)

where *t*_*j*_ is the travel time across link *j*, and *x*_*j*_ is the flow on link *j* in the initial network according to the user equilibrium assignment model [30]. User equilibrium assignment can be performed using *TransCAD*. The system-wide travel time cost $c_{e}^{i}$ can be calculated as follows if the repair link *e* at the current situation after (*i−*1) links are repaired:

$$c_{e}^{i}=\sum_{j\in \left\{E_{ni+e},E_{ri-e}\right\}}t_{j}^{e}x_{j}^{e},$$ (2)

where $t_{j}^{e}$ is the travel time across link *j*, and $x_{j}^{e}$ is the flow on link *j* in the current network according to the user equilibrium assignment model [30]. The user equilibrium assignment model enables the travel time and flow in our study to be consistent with the realistic road network. The critical link can be obtained as follows:

$$I_{e}^{i}=\frac{c_{e}^{i}}{c_{0}}.$$ (3)

The value of $I_{e}^{i}$ for the same road network state, of which the link is the smallest, is the critical link for the current network.

### Algorithm

The objective of our research is to minimize the cumulative whole road network travel cost along with the restoration of the damaged link with only one repair crew. The following assumptions are made before constructing the model: (1) the travel time of the repair crew from one link to another is not considered; (2) damaged links only have two statuses: waiting for repair or return to normal after restoration; and (3) specific repair time for one damaged link is not considered. From these assumptions, for each repair step, our objective function and constraint set are formulated as follows:

$$I_{e}^{i}=\frac{c_{e}^{i}}{c_{0}},$$ (4)

$$s.t.\;\sum_{e}I_{e}^{i}\leq 1,e\in E_{r},$$ (5)

$$\sum_{i}I_{e}^{i}\leq 1,\;i_{e}^{i}\in \left\{0\;,1\right\},$$ (6)

where $I_{e}^{i}$ is denoted as follows:

$$I_{e}^{i}=\{\begin{array}{l} 1\;,\;link\;e\;is\;repaired\;completely\;in\;the\;ith\;step\\ 0\;,\;otherwise \end{array}.$$ (7)

Eq 5 denotes that the repair crew can only repair one link at one step. Eq 6 denotes that any damaged link is rehabilitated at only one step.

Specifically, we hope each repair step of repair crew can reduce whole road network travel cost to the greatest extent. The final repair schedule derived by each step decision is also optimal. In other words, repairing crew make the best choice according to the current state at each step, and the each step best choice make the final global optimal choice as shown in Eq 8. The left side of Eq 8 which is our objectives indicates global optimal solution, repair order is optimization variables. The right side of Eq 8 shows the sum of each local optimal solution. We can achieve global optimal solution just through local optimal choice since Eq 8 is correct. Relevant proof will be given in the next section.

**Fig 1:**
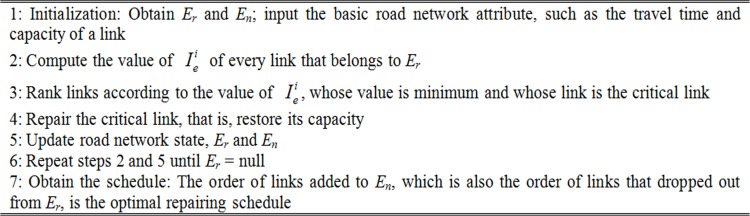
Greedy algorithm procedure.

**Fig 2:**
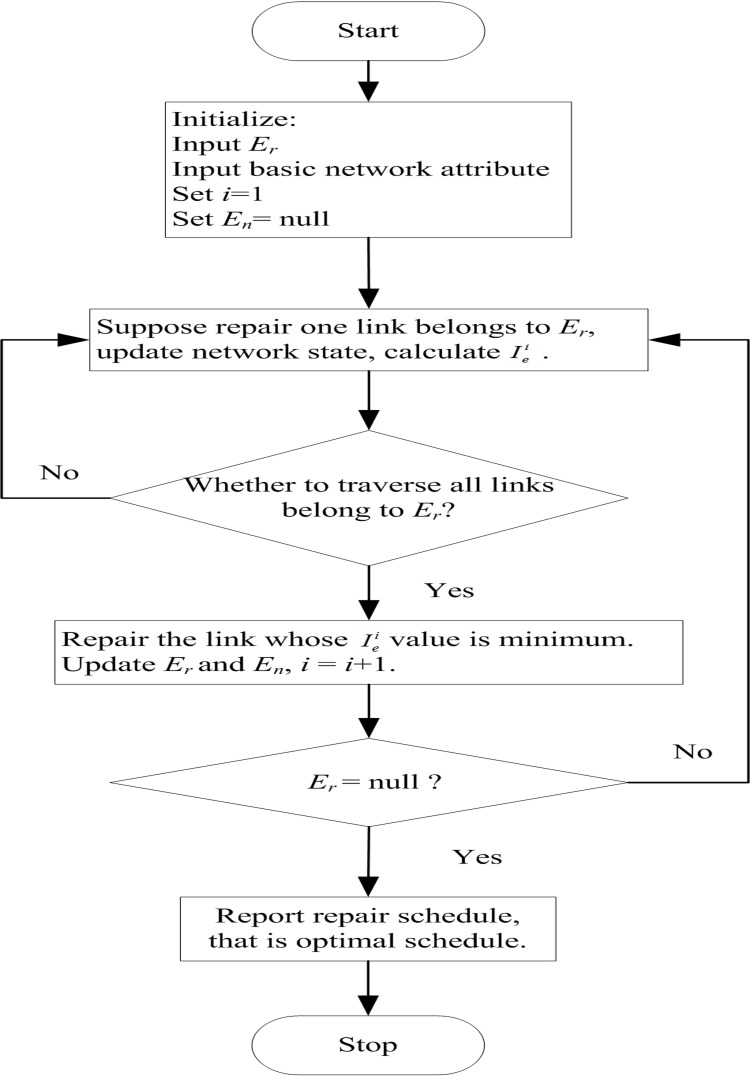
Greedy algorithm flowchart.

The exhaustive search algorithm is clearly feasible in resolving the aforementioned problem, but it will require a considerable amount of time. Therefore, we propose the greedy algorithm to solve the problem. We provide the critical link priority according to the greedy principle. The critical link determined by Eq 4. That means Eq 4 is the selection function, which determined which link to repair each step. We repair the critical link from the rest of *E*_*r*_ until all damage links are restored. However, the ranking of critical links cannot remain unchanged all the time because of the change in road network. Therefore, updating the ranking of critical links after a link restoration is necessary. Fig 1 shows the greedy algorithm. To make it more clear, Fig 2 indicates the greedy algorithm flowchart.

### Proof

In the repair process, the repair crew repairs the critical link, whose $I_{e}^{i}$ is the minimum. The result is a local optimal solution. We must prove that the greedy algorithm to our problem can obtain the global optimal solution through the local optimal solution, which confirms that the following equation is correct:

$$\min\sum_{i=1}^{m}I_{e}^{i}=\sum_{i=1}^{m}\min\left(I_{e}^{i}\right).$$ (8)

The proof consists of two parts. First, the algorithm is proven to produce an urban road network repair schedule. Second, the urban road network repair schedule based on the algorithm is proven optimal. Let *T*_2_ represent the repair schedule produced by the greedy algorithm. Evidently, the urban road network repair schedule problem must have a feasible solution. Accordingly, *T*_2_ is a feasible solution. *T*_2_ is clearly optimal if *E*_*r*_ only contains one link.

The solution *T*_1_ is produced assuming that an optimal algorithm to the problem of urban road network repair crew scheduling is available. *T*_1_ is not equal to *T*_2_, which indicates that the repair order of the two links is opposite, at least between *T*_1_ and *T*_2_. Assuming that *T*_1_: e1→e2→e3→e5→e4→*T*_11_, then *T*_2_: e1→e2→e3→e4→e5→*T*_22_, where *T*_11_ and *T*_22_ represent the repair order of the rest link of *E*_*r*_, except for e1, e2, e3, e4, and e5, which belong to *T*_1_ and *T*_2_, respectively. A solution *T*_3_ for the problem of urban road network repair schedule is thus constructed. *T*_3_ is nearly the same as *T*_1_. The only difference is the repair order of e4 and e5, i.e., *T*_3_: e1→e2→e3→e4→e5→*T*_11_. *T*_3_ is partly the same as *T*_2_. *T*_3_ is clearly a feasible solution.

For *T*_1_:

$$\min\sum_{i=1}^{m}I_{e}^{i}=I_{e1}^{1}+I_{e2}^{2}+I_{e3}^{3}+I_{e5}^{4}+I_{e4}^{5}+A_{1};$$ (9)

For *T*_3_:

$$\sum_{i=1}^{m}I_{e}^{i}=I_{e1}^{1}+I_{e2}^{2}+I_{e3}^{3}+I_{e4}^{4}+I_{e5}^{5}+A_{2};$$ (10)

where $A_{1}=\sum_{i=6}^{m}\left(I_{e}^{i}\right)$ for *T*_1_ and $A_{2}=\sum_{i=6}^{m}\left(I_{e}^{i}\right)$ for *T*_3_.

For *T*_1_:

$$I_{e4}^{5}=\frac{c_{e4}^{5}}{c_{0}},$$ (11)

$$c_{e4}^{5}=\sum_{j\in \left\{E_{n5}+e4,E_{r5}-e4\right\}}t_{j}^{e4}x_{j}^{e4},$$ (12)

$$E_{n5}=E_{n1}+e1+e2+e3+e5.$$ (13)

For *T*_3_:

$$I_{e5}^{5}=\frac{c_{e5}^{5}}{c_{0}},$$ (14)

$$c_{e5}^{5}=\sum_{j\in \left\{E_{n5}+e5,E_{r5}-e5\right\}}t_{j}^{e5}x_{j}^{e5},$$ (15)

$$E_{n5}=E_{n1}+e1+e2+e3+e4.$$ (16)

Therefore, $I_{e4}^{5}=I_{e5}^{5}$. Similarly, *A*_1_ = *A*_2_. $I_{e5}^{4}$ and $I_{e4}^{4}$ are the unique difference between *T*_1_ and *T*_3_ according to the user equilibrium assignment model. The greedy principle determines that the repair order of e4 belongs to *T*_3_. Hence, $I_{e5}^{4}\geq I_{e4}^{4}$, then

$$\left(\min\sum_{i=1}^{m}I_{e}^{i}=I_{e1}^{1}+I_{e2}^{2}+I_{e3}^{3}+I_{e5}^{4}+I_{e4}^{5}+A_{1}\right)\geq \left(\sum_{i=1}^{m}I_{e}^{i}=I_{e1}^{1}+I_{e2}^{2}+I_{e3}^{3}+I_{e4}^{4}+I_{e5}^{5}+A_{2}\right).$$ (17)

*T*_3_ is actually closer to *T*_2_ than *T*_1_. *T*_1_ and *T*_2_ have made *n* different decisions. Similar to constructing *T*_3_, we can obtain *T*_2_ via finite transformation. The value of $\sum_{i=1}^{m}I_{e}^{i}$ is guaranteed to be no more than the value of $\min\sum_{i=1}^{m}I_{e}^{i}$ in translation. The solution of *T*_2_ is essentially $\sum_{i=1}^{m}\min I_{e}^{i}$. Therefore, Eq 8 is correct, and *T*_2_ is optimal. This result implies that *T*_2_ is the optimal urban road network repair schedule.

## Numerical Results

**Fig 3:**
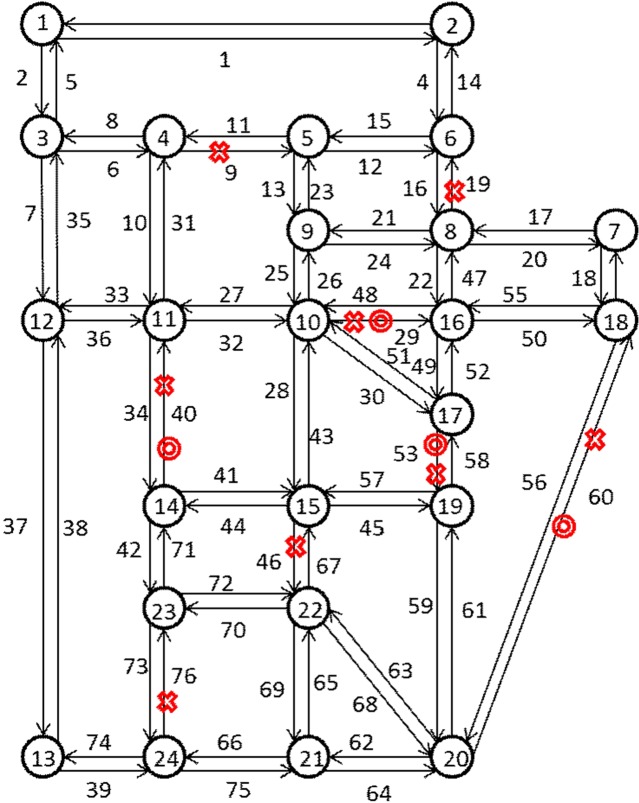
Sioux Falls network.

We propose the optimal schedule for urban road network repair based on the greedy algorithm on the well-known Sioux Falls network (Fig 3), which contains 24 nodes, 76 links, and 576 origin–destination (OD) movements. The Sioux Falls network is abstracted by Chen and Tzeng according to the Northridge earthquake in America [23]. It is a classic experimental network in transport research. The mean OD demand (Table 2), free-flow travel time (Table 3), and network capacity (Table 3) are the same as those used in the research of Li and Ma [31].

**Table 2:** Traffic demand of Sioux Falls network (vehicle/h).

From/To	1	2	3	4	5	6	7	8	9	10	11	12	13	14	15	16	17	18	19	20	21	22	23	24
1	0	100	100	500	200	300	500	800	500	1300	500	200	500	300	500	500	400	100	300	300	100	400	300	100
2	100	0	100	200	100	400	200	400	200	600	200	100	300	100	100	400	200	0	100	100	0	100	0	0
3	100	100	0	200	100	300	100	200	100	300	300	200	100	100	100	200	100	0	0	0	0	100	100	0
4	500	200	200	0	500	400	400	700	700	1200	1400	600	600	500	500	800	500	100	200	300	200	400	500	200
5	200	100	100	500	0	200	200	500	800	1000	500	200	200	100	200	500	200	0	100	100	100	200	100	0
6	300	400	300	400	200	0	400	800	400	800	400	200	200	100	200	900	500	100	200	300	100	200	100	100
7	500	200	100	400	200	400	0	1000	600	1900	500	700	400	200	500	1400	1000	200	400	500	200	500	200	100
8	800	400	200	700	500	800	1000	0	800	1600	800	600	600	400	600	2200	1400	300	700	900	400	500	300	200
9	500	200	100	700	800	400	600	800	0	2800	1400	600	600	600	900	1400	900	200	400	600	300	700	500	200
10	1300	600	300	1200	1000	800	1900	1600	2800	0	4000	2000	1900	2100	4000	4400	3900	700	1800	2500	1200	2600	1800	800
11	500	200	300	1500	500	400	500	800	1400	3900	0	1400	1000	1600	1400	1400	1000	100	400	600	400	1100	1300	600
12	200	100	200	600	200	200	700	600	600	2000	1400	0	1300	700	700	700	600	200	300	400	300	700	700	500
13	500	300	100	600	200	200	400	600	600	1900	1000	1300	0	600	700	600	500	100	300	600	600	1300	800	800
14	300	100	100	500	100	100	200	400	600	2100	1600	700	600	0	1300	700	700	100	300	500	400	1200	1100	400
15	500	100	100	500	200	200	500	600	1000	4000	1400	700	700	1300	0	1200	1500	200	800	1100	800	2600	1000	400
16	500	400	200	800	500	900	1400	2200	1400	4400	1400	700	600	700	1200	0	2800	500	1300	1600	600	1200	500	300
17	400	200	100	500	200	500	1000	1400	900	3900	1000	600	500	700	1500	2800	0	600	1700	1700	600	1700	600	300
18	100	0	0	100	0	100	200	300	200	700	200	200	100	100	200	500	600	0	300	400	100	300	100	0
19	300	100	0	200	100	200	400	700	400	1800	400	300	300	300	800	1300	1700	300	0	1200	400	1200	300	100
20	300	100	0	300	100	300	500	900	600	2500	600	500	600	500	1100	1600	1700	400	1200	0	1200	2400	700	400
21	100	0	0	200	100	100	200	400	300	1200	400	300	600	400	800	600	600	100	400	1200	0	1800	700	500
22	400	100	100	400	200	200	500	500	700	2600	1100	700	1300	1200	2600	1200	1700	300	1200	2400	1800	0	2100	1100
23	300	0	100	500	100	100	200	300	500	1800	1300	700	800	1100	1000	500	600	100	300	700	700	2100	0	700
24	100	0	0	200	0	100	100	200	200	800	600	500	700	400	400	300	300	0	100	400	500	1100	700	0

**Table 3:** Link Parameters.

Link	Link capacity(vehicle/h)	Free-flow travel time (h)
1 and 3	15000	6
2 and 5	10000	2
4 and 14	10000	1.5
6 and 8	10000	2
9 and 11	12500	3.5
12 and 15	15000	3
7 and 35	10000	4
10 and 31	12500	3.5
13 and 23	10000	1.5
25 and 26	10000	1.5
21 and 24	15000	2.5
16 and 19	10000	1
22 and 47	15000	1.5
17 and 20	15000	2.5
18 and 54	15000	1.5
33 and 36	10000	2
27 and 32	15000	3
29 and 48	15000	2.5
50 and 55	15000	2.5
37 and 38	10000	10
34 and 40	10000	4.5
42 and 71	10000	2.5
73 and 76	10000	3.5
41 and 44	15000	3
70 and 72	15000	3
28 and 43	15000	4
46 and 67	15000	2
65 and 69	15000	3
30 and 51	15000	3.5
45 and 57	15000	2.5
63 and 68	15000	4.5
49 and 52	15000	2
53 and 58	15000	2
59 and 61	15000	5.5
56 and 60	15000	10
39 and 74	10000	2
66 and 75	10000	3.5
62 and 64	15000	3

The link capacity reduction range between 80% and 75% is the most appropriate for the test network according to the research of Sullivan et al. [29] and the connectivity of the Sioux Falls network. The two experiments in the test are as follows. The first experiment supposes that eight links are damaged in the Sioux Falls network. We pay attention to the variety of ranking of the critical link. We illustrate our greedy algorithm clearly through the first experiment. The second experiment supposes that four links are damaged in the Sioux Falls network. We provide all 24 repair schedules for comparison. The second experiment proves the correctness of the greedy algorithm with respect to our research objective. The damaged links are random without losing generality.

### The First Experiment

Suppose that links e9, e19, e29, e40, e46, e53, e60, and e74 of the Sioux Falls network (Fig 3) are damaged. The capacity reduction is 80%. We obtain *E*_*r*_ = {e9, e19, e29, e40, e46, e53, e60, e74}, and then calculate the value of $I_{e}^{1}$ for every damaged link that belongs to *E*_*r*_. Table 4 shows that under the circumstances, repair link e40 will enable the repair work gain maximum benefit. After repair link e40, the network state also changes because of the interaction among links. Therefore, we cannot repair link e74 after repairing link e40. We must re-evaluate the relative importance of the damaged links after link e40 restoration. That is, we should calculate the value of $I_{e}^{2}$ for every damaged link that belongs to *E*_*r*2_, and then decide which link to repair. In this case, the link for repair happens to be e74, which is the optimal choice. From this analogy, we can finally obtain the optimal schedule as e40→e74→e53→e46→e29→e19→e9→e60.

**Table 4:** Rank of critical link under different road network states.

link	e9	e19	e29	e53	e40	e46	e60	e74	Ranking of critical link
$I_{e}^{1}$	0.9663	0.9510	0.9594	0.9216	0.8731	0.9626	0.9540	0.9096	e40, e74, e53, e19, e60, e29, e46, e9
$I_{e}^{2}$	0.8538	0.8601	0.8838	0.8818	**✓**	0.8552	0.9240	0.8375	e74, e9, e46, e19, e53, e29, e60
$I_{e}^{3}$	0.8286	0.8262	0.8044	0.7760	**✓**	0.8163	0.8172	**✓**	e53, e29, e46, e60, e19, e9
$I_{e}^{4}$	0.7590	0.7665	0.7690	**✓**	**✓**	0.7481	0.7618	**✓**	e46, e9, e60, e19, e29
$I_{e}^{5}$	0.7298	0.7372	0.7251	**✓**	**✓**	**✓**	0.7466	**✓**	e29, e9, e19, e60
$I_{e}^{6}$	0.7161	0.7092	**✓**	**✓**	**✓**	**✓**	0.7124	**✓**	e19, e60, e9
$I_{e}^{7}$	0.6981	**✓**	**✓**	**✓**	**✓**	**✓**	0.7054	**✓**	e9, e60
$I_{e}^{8}$	**✓**	**✓**	**✓**	**✓**	**✓**	**✓**	0.6880	**✓**	e60

*Note*: **✓**represents the link has been repaired.

The rank of the critical link changes with the road network change are shown in Table 4. Table 4 shows that the rank of the critical link has almost nearly changed after link restoration. The links in the road network are affected by each other one another. We pay attention to focus on the situation after link e40 restoration. The value of $I_{e}^{1}$ is 0.8731. However, the values of $I_{e29}^{2}$, $e_{53}^{2}$, and $e_{60}^{2}$ will be 0.8838, 0.8818, and 0.9240, respectively, if we repair links e29, e53, or e60 subsequently. These values are all greater than 0.8731, indicating that the effect of repairing two links is less than that of one key link. The occurrence of this situation is attributed to the Braess’ paradox. The situation considerably wastes limited repair resources, which should be strongly avoided. In our research, we can predict which link will cause a significantly higher whole network travel cost. With regard to the urban road network repair schedule based on the greedy algorithm, we guarantee that limited repair resources will play the biggest role in each repair stage. The repair schedule is optimal for the current situation, but also the best for the global situation. Our schedule considers link interaction. Therefore, the optimal schedule is e40→e74→e53→e46→e29→e19→e9→e60 if we have only one crew. We can obtain the optimal schedule of e40, e74→e53, e46→e29, e19→e9, e60, rather than recalculate, if we have two crews. In the same manner, we can also directly obtain the optimal schedule if we have three or more crews. That is, our optimal schedule based on only one crew can be expanded.

### The Second Experiment

Suppose that links e29, e40, e53, and e60 in the Sioux Falls network are damaged (Fig 3), the capacity reduction is 80%. According to the greedy algorithm, our optimal schedule is e53→e40→e29→e60 (Table 5). We also obtain all 24 repair crew schedules using the exhaustion method for comparison.

**Table 5:** Greedy algorithm results.

$\min\left(I_{e}^{i}\right)$	$\min\left(I_{e}^{1}\right)$	$\min\left(I_{e}^{2}\right)$	$\min\left(I_{e}^{3}\right)$	$\min\left(I_{e}^{4}\right)$	$\sum_{i=1}^{4}\min\left(I_{e}^{i}\right)$
value	0.917	0.8619	0.8242	0.8123	3.4154
link	e53	e40	e29	e60	—

**Fig 4:**
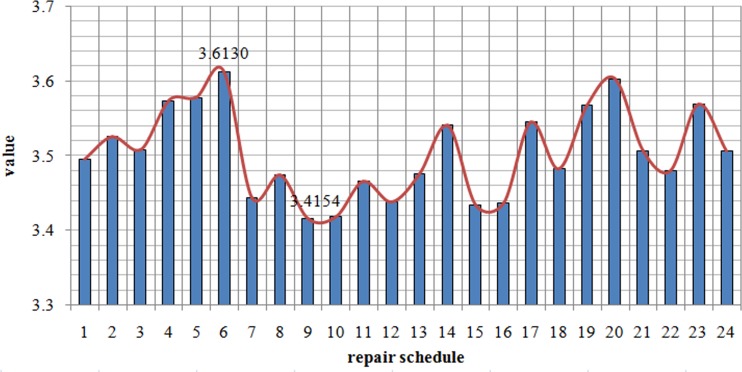
Comparison of different repair schedules.

Fig 4 provides the value $\sum_{i=1}^{4}I_{e}^{i}$ of 24 repair schedules. The column indicates the $\sum_{i=1}^{4}I_{e}^{i}$ value of every repair schedule, the row indicates schedule number. The column clearly shows that the value of $\sum_{i=1}^{4}I_{e}^{i}$ in the 9th repair schedule is the minimum. The 9th repair schedule is the same as the repair schedule based on the greedy algorithm. The result indicates the correctness of Eq 8. The value of $\sum_{i=1}^{4}I_{e}^{i}$ in the 24 repair schedules appears to be less different. However, this value is only the ratio. The difference of the restoration effect will be large if multiplied by the whole road network travel cost for different repair schedules. As shown in Fig 4, the difference between the best and worst schedules remains significant. Therefore, quickly and efficiently obtaining the optimal repair schedule is significant in road network restoration.

## Conclusion

Certain incidents in urban road networks can cause the decline of the capacity of some links, which will lead to traffic congestion or even gridlock, and increase the travel cost of the whole network. Although such events are not as serious as disasters, they happen more frequently, and thus, are more relevant to our life. Only a few studies are related to this topic. We intend to conduct basic research regarding this problem. The core of the problem is how to allocate limited resources to achieve similar goals to those of disaster research. How limited resources can be allocated to minimize the cumulative whole road network travel cost along with the restoration of damaged link is the objective of our research. We define the critical link for our objective, which considers link interaction. Moreover, the link is dynamic. We repair the critical link to quickly achieve our objective based on the greedy algorithm, which aims to obtain the global optimal solution using the local optimal solution. The repair order of the damaged links is the optimal schedule. We prove that the greedy algorithm is applicable to our objective in theory and through a case study.

Our concern is road network restoration. Therefore, the critical links we define are highly suitable for road network repair instead of road network robustness. The link, whose restoration is best for the current road network, will be the critical link. The ranking of the critical link obviously changes because of the interaction among links after a link is repaired. The case study clearly demonstrates this situation. That is, the evaluation of the critical link must be dynamic. The case study also shows that the effect of repairing two links is not always better than the effect of repairing one link because of the Braess’ paradox. If the wrong link is selected for repair, the road network condition will worsen rather than improve. Our research can completely avoid the aforementioned poor decision. The evaluation of the critical link before each repair step fully utilizes the limited resources. Although our optimal schedule assumes that we can only repair one link for every step, the operation can be expanded to repair two or more links for every step rather than recalculate. For example, the optimal schedule is e40→e74→e53→e46→e29→e19→e9→e60 because the case shows that we have only one crew. The optimal schedule is e40, e74→e53, e46→e29, e19→e9, e60 if we have two crews, and so on. Varying solutions are available for the road network repair schedule. The greedy algorithm we apply can obtain the global optimal schedule through the local optimal schedule, which considerably reduces computational complexity and improves computational efficiency. The algorithm is highly efficient even if the road network is extremely large. In addition, it is significant and can be used as a guide in real-life applications.

Actually, greedy algorithm can obtain the global optimal solution through the local optimal solution thus reduces computational complexity and improves computational efficiency. However, not all problems can obtain global optimal solution through greedy algorithm. Therefore, we have proved that theoretically in section 2. The second experiment also proved it. Our optimal schedule has some limitations. The specific repair time of different damaged links and the time the crew travels from one damaged link to another are not considered. However, these issues are essential in real life. Consequently, the optimal schedule obtained using our proposed technique cannot be directly applied to real–life situations. These issues require further investigation. Combining the current research results with practical issues can be a worthwhile direction for future research.

## References

[1] Corley HW, Sha DY. Most vital links and nodes in weighted networks. Operations Research Letters. 1982; 1: 157–160.

[2] Nardelli E, Proietti G, Widmayer P. Finding the detour-critical edge of a shortest path between two nodes Information Processing Letters. 1998; 67: 51–54.

[3] Scott DM, Novak DC, Aultman-Hall L, Guo F. Network Robustness Index: A new method for identifying critical links and evaluating the performance of transportation networks. Journal of Transport Geography. 2006; 14: 215–227.

[4] Oliveira ELD, Portugal LDS, Junior WP. Determining Critical Links in a Road Network: Vulnerability and Congestion Indicators Procedia—Social and Behavioral Sciences. 2014; 162: 158–167.

[5] Rupi F, Angelini S, Bernardi S, Danesi A, Rossi G. Ranking Links in a Road Transport Network: A Practical Method for the Calculation of Link Importance Transportation Research Procedia. 2015; 5: 221–232.

[6] Hou LW, Jiang F. Study on the Relative Importance of Links in Urban Roads Network. Systems Engineering-Theory Methodology Application. 2004; 13: 425–428.

[7] Sohn J. Evaluating the significance of highway network links under the flood damage: An accessibility approach. Transportation Research Part A Policy & Practice. 2006; 40: 491–506.

[8] Gao ZK, Jin ND. A directed weighted complex network for characterizing chaotic dynamics from time series. Nonlinear Analysis Real World Applications. 2012; 13: 947–952.

[9] Gao ZK, Yang YX, Fang PC, Jin ND, Xia CY, Hu LD. Multi-frequency complex network from time series for uncovering oil-water flow structure. Scientific Reports. 2015; 5:10.1038/srep08222 PMC4316157 25649900

[10] Gao ZK, Fang PC, Ding MS, Jin ND. Multivariate weighted complex network analysis for characterizing nonlinear dynamic behavior in two-phase flow. Experimental Thermal & Fluid Science. 2015; 60: 157–164.

[11] Iyer S, Killingback T, Sundaram B, Wang Z. Attack Robustness and Centrality of Complex Networks. Plos One. 2013; 8:10.1371/journal.pone.0059613 PMC3615130 23565156

[12] Matisziw TC, Murray AT. Modeling s-t Path Availability to Support Disaster Vulnerability Assessment of Network Infrastructure. Computers & Operations Research. 2009; 36: 16–26.

[13] Matisziw T, Murray A, Grubesic T. Exploring the vulnerability of network infrastructure to disruption. The Annals of Regional Science. 2009; 43: 307–321.

[14] Scaparra MP, Church RL. A bilevel mixed-integer program for critical infrastructure protection planning. Computers & Operations Research. 2008; 35: 1905–1923.

[15] Aliakbarian N, Dehghanian F, Salari M. A bi-level programming model for protection of hierarchical facilities under imminent attacks. Computers & Operations Research. 2015; 64: 210–224.

[16] Hu F, Yeung CH, Yang S, Wang W, Zeng A. Recovery of infrastructure networks after localised attacks. Scientific Reports. 2016; 6:10.1038/srep24522 PMC4830952 27075559

[17] Matisziw TC, Murray AT, Grubesic TH. Strategic Network Restoration. Networks & Spatial Economics. 2010; 10:

[18] Bonyuet M, GarciaDIAZ A, Hicks I. Optimization procedures for simultaneous road rehabilitation and bridge replacement decisions in highway networks. Engineering Optimization. 2001; 34: 445–459.

[19] Aksu DT, Ozdamar L. A mathematical model for post-disaster road restoration: Enabling accessibility and evacuation. Transportation Research Part E Logistics & Transportation Review. 2014; 61: 56–67.

[20] Zhang JH, Li J, Liu ZP. Multiple-resource and multiple-depot emergency response problem considering secondary disasters. Expert Systems with Applications. 2012; 39: 11066–11071.

[21] Özdamar L, Aksu DT, Ergüneş B. Coordinating debris cleanup operations in post disaster road networks. Socio-Economic Planning Sciences. 2014; 48: 249–262.

[22] Duque PAM, Dolinskaya IS, Sörensen K. Network repair crew scheduling and routing for emergency relief distribution problem. European Journal of Operational Research. 2015; 248:

[23] Chen YW, Tzeng GH. A Fuzzy Multi-objective Model for Reconstructing the Post-quake Road-network by Genetic Algorithm. International Journal of Fuzzy Systems. 1999:

[24] Fiedrich F, Gehbauer F, Rickers U. Optimized resource allocation for emergency response after earthquake disasters. Safety Science. 2000; 35: 41–57.

[25] Feng C-M, Wang T-C. Highway emergency rehabilitation scheduling in post-earthquake 72 hours. Journal of the 5th Eastern Asia Society for Transportation Studies. 2003; 5: 3276–3285.

[26] Chang FS, Wu JS, Lee CN, Shen HC. Greedy-search-based multi-objective genetic algorithm for emergency logistics scheduling. Expert Systems with Applications. 2014; 41: 2947–2956.

[27] Ying KC, Lin SW, Huang CY. Sequencing single-machine tardiness problems with sequence dependent setup times using an iterated greedy heuristic. Expert Systems with Applications. 2009; 36: 7087–7092.

[28] Ying KC, Cheng HM. Dynamic parallel machine scheduling with sequence-dependent setup times using an iterated greedy heuristic. Expert Systems with Applications. 2010; 37: 2848–2852.

[29] Sullivan JL, Novak DC, Aultman-Hall L, Scott DM. Identifying critical road segments and measuring system-wide robustness in transportation networks with isolating links: A link-based capacity-reduction approach. Transportation Research Part A Policy & Practice. 2010; 44: 323–336.

[30] Wardrop JG Some theoretical aspects of road traffic research. In: Proceeding of the Institution of the Institution of Civil Eng, 1952. pp 72–73

[31] Li SL, Ma ZJ. User Equilibrium-based Post-earthquake Relief Routing Problems under Traffic Control. Journal of Industrial Engineering & Engineering Management. 2014; 28: 148–155.

